# Modulation of Genetic Associations with Serum Urate Levels by Body-Mass-Index in Humans

**DOI:** 10.1371/journal.pone.0119752

**Published:** 2015-03-26

**Authors:** Jennifer E. Huffman, Eva Albrecht, Alexander Teumer, Massimo Mangino, Karen Kapur, Toby Johnson, Zoltán Kutalik, Nicola Pirastu, Giorgio Pistis, Lorna M. Lopez, Toomas Haller, Perttu Salo, Anuj Goel, Man Li, Toshiko Tanaka, Abbas Dehghan, Daniela Ruggiero, Giovanni Malerba, Albert V. Smith, Ilja M. Nolte, Laura Portas, Amanda Phipps-Green, Lora Boteva, Pau Navarro, Asa Johansson, Andrew A. Hicks, Ozren Polasek, Tõnu Esko, John F. Peden, Sarah E. Harris, Federico Murgia, Sarah H. Wild, Albert Tenesa, Adrienne Tin, Evelin Mihailov, Anne Grotevendt, Gauti K. Gislason, Josef Coresh, Pio D'Adamo, Sheila Ulivi, Peter Vollenweider, Gerard Waeber, Susan Campbell, Ivana Kolcic, Krista Fisher, Margus Viigimaa, Jeffrey E. Metter, Corrado Masciullo, Elisabetta Trabetti, Cristina Bombieri, Rossella Sorice, Angela Döring, Eva Reischl, Konstantin Strauch, Albert Hofman, Andre G. Uitterlinden, Melanie Waldenberger, H-Erich Wichmann, Gail Davies, Alan J. Gow, Nicola Dalbeth, Lisa Stamp, Johannes H. Smit, Mirna Kirin, Ramaiah Nagaraja, Matthias Nauck, Claudia Schurmann, Kathrin Budde, Susan M. Farrington, Evropi Theodoratou, Antti Jula, Veikko Salomaa, Cinzia Sala, Christian Hengstenberg, Michel Burnier, Reedik Mägi, Norman Klopp, Stefan Kloiber, Sabine Schipf, Samuli Ripatti, Stefano Cabras, Nicole Soranzo, Georg Homuth, Teresa Nutile, Patricia B. Munroe, Nicholas Hastie, Harry Campbell, Igor Rudan, Claudia Cabrera, Chris Haley, Oscar H. Franco, Tony R. Merriman, Vilmundur Gudnason, Mario Pirastu, Brenda W. Penninx, Harold Snieder, Andres Metspalu, Marina Ciullo, Peter P. Pramstaller, Cornelia M. van Duijn, Luigi Ferrucci, Giovanni Gambaro, Ian J. Deary, Malcolm G. Dunlop, James F. Wilson, Paolo Gasparini, Ulf Gyllensten, Tim D. Spector, Alan F. Wright, Caroline Hayward, Hugh Watkins, Markus Perola, Murielle Bochud, W. H. Linda Kao, Mark Caulfield, Daniela Toniolo, Henry Völzke, Christian Gieger, Anna Köttgen, Veronique Vitart

**Affiliations:** 1 Medical Research Council (MRC) Human Genetics Unit, MRC Institute of Genetics and Molecular Medicine (IGMM), University of Edinburgh, Edinburgh, United Kingdom; 2 Institute of Genetic Epidemiology, Helmholtz Zentrum München—German Research Center for Environmental Health, Neuherberg, Germany; 3 Interfaculty Institute for Genetics and Functional Genomics, Ernst-Moritz-Arndt-University Greifswald, Greifswald, Germany; 4 King's College London, St. Thomas' Hospital Campus, London, United Kingdom; 5 Department of Medical Genetics, University of Lausanne, Lausanne, Switzerland; 6 Swiss Institute of Bioinformatics, Lausanne, Switzerland; 7 William Harvey Research Institute, Barts and The London School of Medicine and Dentistry, Queen Mary University of London, London, United Kingdom; 8 Institute for Maternal and Child Health—Istituto Di Ricovero e Cura a Carattere Scientifico (IRCCS) "Burlo Garofolo", Trieste, Italy; 9 University of Trieste, Trieste, Italy; 10 Division of Genetics and Cell Biology, San Raffaele Scientific Institute, Milano, Italy; 11 Department of Psychology, The University of Edinburgh, Edinburgh, United Kingdom; 12 Centre for Cognitive Ageing and Cognitive Epidemiology, The University of Edinburgh, Edinburgh, United Kingdom; 13 Estonian Genome Center, University of Tartu, Tartu, Estonia; 14 Broad Institute, Cambridge, MA, United States of America; 15 Children’s Hospital Boston, Boston, MA, United States of America; 16 Department of Chronic Disease Prevention, National Institute for Health and Welfare (THL), Helsinki, Finland; 17 Department of Cardiovascular Medicine, Wellcome Trust Centre for Human Genetics, University of Oxford, Oxford, United Kingdom; 18 Department of Epidemiology, Johns Hopkins Bloomberg School of Public Health, Baltimore, MD, United States of America; 19 Clinical Research Branch, National Institute on Aging, Baltimore, MD, United States of America; 20 Member of Netherlands Consortium for Healthy Aging (NCHA) sponsored by Netherlands Genomics Initiative (NGI), Leiden, The Netherlands; 21 Department of Epidemiology, Erasmus Medical Center, Rotterdam, The Netherlands; 22 Institute of Genetics and Biophysics "A. Buzzati-Traverso"—Consiglio Nazionale delle Ricerche (CNR), Naples, Italy; 23 Biology and Genetics section, Department of Life and Reproduction Sciences, University of Verona, Verona, Italy; 24 Icelandic Heart Association Research Institute, Kopavogur, Iceland; 25 University of Iceland, Reykjavik, Iceland; 26 Department of Epidemiology, University Medical Center Groningen, University of Groningen, Groningen, The Netherlands; 27 Institute of Population Genetics, National Research Council of Italy, Sassari, Italy; 28 Department of Biochemistry, University of Otago, Dunedin, New Zealand; 29 Uppsala Clinical Research Center, Uppsala University Hospital, Upsalla, Sweden; 30 Department of Immunology, Genetics and Pathology, Rudbeck Laboratory, Uppsala University, Uppsala, 751 85, Sweden; 31 Center for Biomedicine, European Academy Bozen/Bolzano (EURAC), Bolzano, Italy; Affiliated Institute of the University of Lübeck, Lübeck, Germany; 32 Faculty of Medicine, University of Split, Croatia, Soltanska 2, Split, 21000, Croatia; 33 Institute of Population Health Sciences and Informatics, University of Edinburgh, Edinburgh, Scotland, United Kingdom; 34 Roslin Institute, The University of Edinburgh, Edinburgh, United Kingdom; 35 Institute of Clinical Chemistry and Laboratory Medicine, University Medicine Greifswald, Ernst-Moritz-Arndt University Greifswald, Greifswald, Germany; 36 Welch Center for Prevention, Epidemiology and Clinical Research, John Hopkins University, Baltimore, MD, United States of America; 37 Department of Medicine, Internal Medicine, Lausanne University Hospital, Lausanne, Switzerland; 38 Tallinn University of Technology, Department of Biomedical Engineering, Chair of Medical Physics, Tallinn, Estonia; 39 Centre of Cardiology, North Estonia Medical Centre, Tallinn, Estonia; 40 Institute of Epidemiology II, Helmholtz Zentrum München—German Research Center for Environmental Health, Neuherberg, Germany; 41 Institute of Epidemiology I, Helmholtz Zentrum München—German Research Center for Environmental Health, Neuherberg, Germany; 42 Research Unit of Molecular Epidemiology, Helmholtz Zentrum München—German Research Center for Environmental Health, Neuherberg, Germany; 43 Institute of Medical Informatics, Biometry and Epidemiology, Chair of Genetic Epidemiology, Ludwig-Maximilians-University, Munich, Germany; 44 Klinikum Grosshadern, Munich, Germany; 45 Bone and Joint Research Group, Department of Medicine, University of Auckland, Auckland, New Zealand; 46 Department of Medicine, University of Otago, Christchurch, New Zealand; 47 Department of Psychiatry/EMGO Institute, VU University Medical Centre, Amsterdam, the Netherlands; 48 Laboratory of Genetics, National Institute on Aging (NIA), Baltimore, MD, United States of America; 49 Department of Chronic Disease Prevention, National Institute for Health and Welfare (THL), Turku, Finland; 50 University Hospital Regensburg, Regensburg, Germany; 51 Department of Medicine, Nephrology Division, Lausanne University Hospital, Lausanne, Switzerland; 52 Max Planck Institute of Psychiatry, Munich, Germany; 53 Institute for Community Medicine, University Medicine Greifswald, Greifswald, Germany; 54 Human Genetics, Wellcome Trust Sanger Institute, Hinxton, United Kingdom; 55 University of Helsinki, Institute of Molecular Medicine, Helsinki, Finland; 56 Department of Mathematics and Informatics, Università di Cagliari, Cagliari, Italy; 57 Department of Statistics, Universidad Carlos III de Madrid, Madrid, Spain; 58 Queen Mary, University of London, London, United Kingdom; 59 Department of Psychiatry, Leiden University Medical Center, Leiden, The Netherlands; 60 Department of Epidemiology, Subdivision Genetic Epidemiology, Erasmus MC, Rotterdam, The Netherlands; 61 Department of Internal Medicine, Erasmus MC, Rotterdam, The Netherlands; 62 Institute of Internal Medicine, Renal Program, Columbus-Gemelli University Hospital, Catholic University, Rome, Italy; 63 on behalf of PROCARDIS; Department of Cardiovascular Medicine, Wellcome Trust Centre for Human Genetics, University of Oxford, Oxford, United Kingdom; 64 University Institute of Social and Preventive Medicine, Lausanne, Switzerland; 65 Renal Division, Freiburg University Hospital, Freiburg, Germany; 66 Medical Genetics Section, University of Edinburgh Centre for Genomics and Experimental Medicine and MRC Institute of Genetics and Molecular Medicine, Edinburgh, United Kingdom; Case Western Reserve University, UNITED STATES

## Abstract

We tested for interactions between body mass index (BMI) and common genetic variants affecting serum urate levels, genome-wide, in up to 42569 participants. Both stratified genome-wide association (GWAS) analyses, in lean, overweight and obese individuals, and regression-type analyses in a non BMI-stratified overall sample were performed. The former did not uncover any novel locus with a major main effect, but supported modulation of effects for some known and potentially new urate loci. The latter highlighted a SNP at *RBFOX3* reaching genome-wide significant level (effect size 0.014, 95% CI 0.008-0.02, P_inter_= 2.6 x 10^-8^). Two top loci in interaction term analyses, *RBFOX3* and *ERO1LB-EDARADD*, also displayed suggestive differences in main effect size between the lean and obese strata. All top ranking loci for urate effect differences between BMI categories were novel and most had small magnitude but opposite direction effects between strata. They include the locus *RBMS1-TANK* (men, P_difflean-overweight_= 4.7 x 10^-8^), a region that has been associated with several obesity related traits, and *TSPYL5* (men, P_difflean-overweight_= 9.1 x 10^-8^), regulating adipocytes-produced estradiol. The top-ranking known urate loci was *ABCG2*, the strongest known gout risk locus, with an effect halved in obese compared to lean men (P_difflean-obese_= 2 x 10^-4^). Finally, pathway analysis suggested a role for N-glycan biosynthesis as a prominent urate-associated pathway in the lean stratum. These results illustrate a potentially powerful way to monitor changes occurring in obesogenic environment.

## Introduction

Epidemiological studies have associated hyper- and hypo-uricemia with multiple common diseases and conditions in humans [1]; hyperuricemia clusters with all metabolic syndrome components and is a causal risk factor for gout development. To date, 28 loci have been identified and replicated accounting for about 7% of the inter-individual variation in age and sex adjusted serum urate (SU) levels [2]. The top two loci, which account for about half of the genetic variance explained so far, have been noted to display marked gender differences in their effect [3–6], while other urate loci not [2,7]. Variants in the solute carrier *SLC2A9* (also known as *GLUT9*) gene have doubled the effect on SU in women (0.40 standard deviation (sd) in [7]) than that observed in men, and variants in the transporter *ABCG2* gene have a stronger effect in men than in women (0.22 sd versus 0.14 sd in [7]).

Body mass index (BMI) is strongly positively correlated with SU levels in population-based studies (phenotypic correlations ranging from 0.27 to 0.44 [8–12]), and the relationship is approximately linear ([12] and S1 Fig.). Obesity is the strongest modifiable risk factor for hyperuricemia and gout [13]. We investigated here to what extent the genetic variants affecting SU are modulated by BMI. The fact that the genetic variants with the largest effect on SU levels are observed in genes encoding for ion transport proteins provides a biological rationale, since the activity of those transporters may be directly or indirectly affected by the metabolic changes associated with BMI increase, e.g. by levels of serum phosphate and hepatic ATP both reported to be inversely correlated with BMI [14,15]. Additionally, many of the newly discovered urate loci are in genes concerned with regulation of energy metabolism and glucose flux which are affected by BMI status. In 2008, a study had suggested that *SLC2A9* variants’ effects on SU may be stronger in severely obese individuals (defined as BMI > 40), with a stronger modulating BMI effect in men than in women [9], while a recent publication suggests the opposite, in a predominantly women study [16]. Both these studies had modest sample sizes, calling for a larger study to be carried out.

Here, we performed a genome-wide investigation for genetic variants influencing serum urate levels in a BMI-dependent fashion, primarily by analysing genome-wide association study (GWAS) stratified by BMI. Stratified analyses are best suited when main effects are very different in magnitude or direction between strata and if the environment factor measured on a continuous scale is not acting linearly. In a discovery set, totalling 41,832 participants, GWAS for SU were performed after stratifying subjects by BMI status categorized into three levels: lean (BMI < 25 kg/m^2^), overweight (25 ≤ BMI ≤ 30) and obese (BMI > 30 kg/m^2^). This allowed investigation of whether stratification revealed new genetic variants influencing SU and to systematically test differences in effects between BMI strata. Interaction between allelic effect and BMI was also investigated using a linear model with introduction of an interaction term and replication attempted in an independent set.

## Materials and Methods

### Study subjects

The discovery BMI-stratified genome-wide association study meta-analyses (GWAMA) combined data from 22 population cohorts encompassing 42741 individuals with measured circulating urate levels and BMI. With six additional follow-up studies, all were studies of European descent participants that contributed to the Global Urate and Gout consortium (GUGC) and have thus been previously described in detail [2]. The study-specific descriptions are reported in S1 Table, in effect a subset of the GUGC publication.

Two extra studies, the Rotterdam study (described in S1 Table as also a GUGC participant) and a New-Zealand study of individuals from Polynesian descent [17] only contributed to the replication for the *CLK4* locus. Sample sizes for the different sub-analyses performed and urate summary statistics for all studies with break down per BMI and gender stratum are detailed in S2 Table.

### Genotype collection

Genome-wide SNP genotyping was undertaken by each cohort using various platforms as previously described [2] and reported in S3 Table. Imputation of allele dosage of SNPs typed in the HapMap CEU population was performed using either MACH or IMPUTE with parameters and pre-imputation filters specified in S3 Table.

### Statistical analysis

#### BMI-stratified main effect GWAMA

Combined-gender and gender-separate association analyses were performed as described in Kolz *et al*. [7] within three body mass index (BMI) categories (nine sub-analyses performed in total): lean (BMI<25), overweight (25≤BMI≤30) and obese (BMI>30). Urate level (mg/dl) was adjusted for age, sex, and if required, ancestry principal components. Medications were not taken into account. Residuals were standardised using a z-score and used as response variable. Genome-wide association analyses were performed using imputed allele doses as predictor variable in linear models, and each study submitted regression summary statistics for meta-analysis. Studies with related individuals used a linear mixed model that additionally accounts for a polygenic random effect (e.g a score test mmscore [18] implemented in the GenABEL package [19]). Softwares used by the different studies to implement association testing are reported in S3 Table.

The results from all individual GWA scans were combined into a fixed-effects meta-analysis using inverse variance weighting, implemented in the MetABEL R package [15]. From individual-study analysis, SNPs with minor allele frequency less than 1% or low imputation quality (assessed by the metrics r2hat (MACH) <0.3 or. info (IMPUTE) <0.4) were excluded. The QQ plots for association statistics from each study were visualised in R. This highlighted that two many results from the PROCARDIS-women dataset departed from the null hypothesis distribution, and this subset was removed from the final meta-analysis as driving many significant results if non-excluded. Study-specific genomic control inflation factors are reported in S4 Table. In the meta-analyses, each individual study results were adjusted using the inflation factors; the overall meta-analysis effects’ standard errors and p-value reported were not further corrected. The overall inflation factor for the nine stratified GWAMA were 1.0167 (lean-combined-gender), 1.0069 (lean-women), 1.0120 (lean-men), 1.0362 (overweight-combined-gender), 1.0167 (overweight-women), 1.0232 (overweight-men), 1.0157 (obese-combined-gender), 1.0180 (obese-women) and 1.0157 (obese-men). The conventional genome-wide significance threshold of 5x10^-8^ was used. To avoid results driven by one or two populations that are likely to be spurious, meta-analysis results for the lower allele frequency variants (MAF <5%) are reported only if at least four populations contributed and if the contribution of any single study as calculated by the R package “meta” (http://cran.r-project.org/) was not greater than 30%. Annotation to known GWAS hits in the vicinity (window of 150 kb centred on index SNP) of novel potential urate loci was made using the NHGRI GWAS catalogue [20], 29-10-2013 update.

#### Main effect gene-based test

A gene-based test for SU association in the BMI-stratified GWAMA was conducted using the VEGAS software. Briefly, this method assigns SNPs to genes (+/- 50kb of 5’ and 3’ UTRs) and combines the association P-values accounting for linkage disequilibrium between markers assigned to the same gene. Analyses were conducted for each of the nine BMI/gender categories GWAMA results. As 17,787 genes are tested, the Bonferroni-corrected threshold for significance is set at 2.8 10^-6^.

Replication of the differential effect of the *CLK4* variant rs7711186 was sought in six independent studies of individuals of European descent, totalling 1259 individuals, in which the marker was either genotyped or well imputed and, as exploratory foray, in a small sample of individuals of Polynesian descent (N = 290) with prevalent obesity.

#### Testing for differences in main effect between BMI strata

The meta-analysed SNP main effects on SU were compared between all pairwise BMI categories (lean-obese, lean-overweight, and overweight-obese) using a *t*-test. Test statistics were calculated using the statistic *t* = (β_bmicat1_ - β_bmicat2_)/sqrt(SE_bmicat1_^2^ + SE_bmicat2_^2^-2r(SE _bmicat1,_ SE _bmicat2_)), with β_bmicat_ and SE_bmicat_ the meta-analysed SNP effect-estimates and their corresponding standard errors, and r the Spearman rank correlation coefficient between meta-analyzed beta-estimates, in each of the BMI categories compared, across all SNPs. Under the null hypothesis of no difference in effect sizes between BMI strata, the t statistic is expected to follow a Student’s t distribution.

### Interaction effect GWAMA

#### Discovery studies

Combined-gender and sex-stratified SNP by BMI interaction analyses were also performed in participating discovery studies using linear regression methods.

Urate residuals were generated using the same covariates and standardisation as described for the stratified main effect GWAS. For studies with related individuals, relatedness was accounted for by fitting ancestry principal components (PCs) derived from the genomic relationship matrix rather than fitting it in full within a mixed model for the association test as the iterative processes used for parameter estimations of the mixed models often did not converge in a pilot run using family-based populations. The number of PCs to account for, varying from study to study and best determined by examination of scree plots (point to which additional PCs all contribute the same percentage of genetic variation), was left to the decision of each study analyst. Each study GWAS was performed on imputed genotype dose using the following model: z(residual)~μ+β_1_BMI+β_2_SNP+β_12_BMI*SNP+ɛ, with BMI as continuous variable, z(residual) the serum urate level adjusted for age, sex (in the combined gender analysis) and ancestry principal components expressed as z-score ((individual trait value minus population mean)/population standard deviation), β the regression coefficients for the fitted effects, ɛ the error term with normal probability distribution.

Softwares used by the different studies to implement association testing are reported in S2 Table. Coefficients estimates for the main effect (β_1_ and β_2_) were not reported for studies that used Quicktest, as this later only reported the interaction term (β_12_). Meta-analyses of the interaction effects (β_12_) coefficients were carried out using MetABEL as described for the stratified main effect GWAS, with a higher MAF cut-off (5%) for each individual study. To avoid results predominantly driven by one population that are likely to be spurious, meta-analysis results with individual study contribution greater than 50% as calculated by the meta R package were filtered out.

As the individual studies genomic control inflation factors (λ) for these analyses were often high (S3 Table), only the studies with a λ less than 1.2 were analysed and sensitivity analyses with a reduced set of studies with λ less than 1.05 were also performed. The overall inflation factors for the GWAMA of interaction terms with the studies with a λ less than 1.2 corrected using genomic control were 0.992 in the combined-gender, 1.011 in the women and 1.024 in the men analyses.

#### Follow-up set

A small number of studies were available for follow-up of the linear interaction analysis, totalling 9298 participants (INGI-Cilento, OGP Talana, NESDA, INCIPE, INGI-FVG and AGES). All follow-up studies analyses were carried out in the combined-gender data-set only and use the “model-robust method” that is implemented in the ProbABEL and Quicktest packages. Application of the model-robust method in principle leads to lower genomic control inflation for the interaction term [21]. To increase sample size in the follow-up, the CoLaus study (N = 5411) was added as a follow-up rather than discovery set for the regression based interaction term analysis. One study (INCIPE-N = 940) had high λ for both main and interaction effects (S3 Table), and was not included in the meta-analysis.

Meta-analyses of the interaction effects (β_12_) coefficients were carried out using MetABEL as previously described for the discovery cohorts. The overall inflation coefficient for this follow-up meta-analysis was 1.018 and 1.006 for the combined discovery and follow-up studies interaction term meta-analysis.

### Pathway Analysis

The pathway analysis was carried out using a SNP-based circular permutation method implemented in an extension of the R package “genomicper” (http://cran.r-project.org/) available upon request to the package’s authors. After lift over to build37, SNPs were annotated to genes when they were located within gene regions using annotations from the NCBI Gene database (http://www. ncbi.nlm.nih.gov/gene; build.37.1) and the SNPs (and associated GWAMA-p-values) were ordered according to their location in the genome. Pathways (n = 229) were downloaded using KEGG.db (http://www. genome.jp/kegg/) and the SNPs and genes assigned to the pathways. SNPs with GWAMA p-values less or equal to 5% were considered associated with trait and associated SNPs within a pathway counted. This count was compared to the distribution of counts obtained from 10,000 circular permutations of the SNPs’ GWAMA association p-values with respect to the SNPs locations. In circular genomic permutation the genome is considered circular and ordered from chromosome 1 to 22 and restarting at chromosome 1 [22]. Each permutation is akin to the spinning of a wheel with the whole starting set of SNP labels and locations fixed at the outside of the wheel and the SNPs’ GWAMA p-values on the rotating wheel. As the SNPs’ p-values rotate to the same degree, they retain patterns of correlation similar to those in the original data. The empirical p-value for the trait-pathway association was calculated from the ratio of the total number of permutations with more significant SNPs than the non-permuted set divided by the total number of permutations performed in the analysis [22].

### Ethics Statements

Participants gave written informed consent to each original study. All studies received approval from their local ethics committees as listed. S1 Table and protocols comply with the tenets of the Declaration of Helsinki.

## Results

### BMI stratified urate GWAS

All 22 participating studies had previously contributed to non-stratified SU analyses [2] and study-specific information is reported in S1 and S3 Tables. All study participants were of European ancestry and displayed BMI distribution typical of that of populations that adopted westernised diet and culture, with more than half of the participants overweight or obese (Table 1). The smallest stratum analysed comprised 4,613 individuals (obese-men category), the largest 17,078 (overweight-all category). Individual study SU descriptive statistics are reported in S2 Table. The median population mean SU per stratum analysed was, as expected from the wealth of epidemiological data, higher in males than females and increasing from the lean to the obese group (Table 1).

**Table 1:** Statistics for the discovery studies mean serum urate levels.

	Lean (BMI < 25 kg/m^2^)		Overweight (BMI 25–30kg/m^2^)		Obese (BMI >30 kg/m^2^)	
	median; Min-Max	N	median; Min-Max	N	median; Min-Max	N
All	4.58; 4.18–5.71	14504	5.36; 4.75–6.19	17078	5.81; 4.98–6.64	9445
Men	5.5; 4.86–6.19	5529	5.9; 5.2–6.72	10058	6.53; 5.31–7.23	4613
Women	4.1; 3.73–4.85	9753	4.6; 4.14–5.41	7189	5.22; 4.63–6.19	4690

Median, Minimum and Maximum values for the mean serum urate (SU) concentrations (mg/dl) amongst the twenty two studies used in the BMI and gender stratified meta-analyses are displayed. N represents the total number of participants analysed in each category.

The stratification process did not yield any novel genome-wide significant signal at the SNP level (P < 5 x 10^-8^) and all but three (*LRRC16*, *SLC16A9* and *RREB1*) of the eleven loci reported in two earlier, non-stratified, SU genome-wide association meta-analyses (GWAMA) of size roughly comparable to the present analyses [7,23], reached genome-wide significance in at least one of the nine strata (Table 2). All other loci encompassing SNP variant(s) with an association P-value below the suggestive threshold of 10^-5^ in any of the nine meta-analyses are listed in S5 Table. Three of these suggestive loci, *A1CF* (lean-combined-gender), *HLF* (obese-combined-gender) and *NRG4* (obese-men) are among the 18 novel, validated and replicated, urate loci in a large recent SU GWAMA (N>140,000 individuals, a subset of which is analysed here) [2]. No functional link with urate homeostasis is obvious from the genes within the other suggestive signals apart potentially for *SLC28A1* (lean-men category), encoding a sodium/nucleoside co-transporter present in kidney. *MYO18D* and *ADAMST17* (both in lean stratum signals) have been previously listed as suggestive loci for serum urate levels in a small study of African American participants [24].

**Table 2:** Loci significantly associated with serum urate within any BMI stratum analysed and mean effect sizes across strata.

				Lean (BMI < 25 kg/m^2^)				Overweight (BMI 25–30 kg/m^2^)				Obese (BMI >30 kg/m^2^)				Effect size Comparison		
Locus	SNP ^a^	A1	A2	fqA1	β_lean_	s.e.	P	fqA1	β_ov_	s.e.	P	fqA1	β_ob_	s.e.	P	P-value 2-sided test
Combined-gender				N = 14504				N = 17078				N = 9445				lean-ov	lean-ob	ov-ob
*SLC2A9*	rs7680126	G	A	0.22	-0.341	0.014	**2.36E-134**	0.22	-0.271	0.013	**2.96E-92**	0.21	-0.298	0.018	**6.38E-62**	NA	NA	NA
*ABCG2*	rs2231142	G	T	0.89	-0.223	0.020	**1.55E-29**	0.89	-0.215	0.019	**1.55E-30**	0.89	-0.135	0.025	1.18E-07	NA	NA	NA
*SLC17A1*;*SLC17A4*	rs1165209	G	A	0.46	-0.085	0.012	**2.98E-13**	0.46	-0.086	0.011	**3.79E-15**	0.46	-0.051	0.015	6.73E-04	9.35E01	7.05E-02	5.39E-02
*SLC22A11*	rs2078267	C	T	0.49	0.064	0.012	7.13E-08	0.49	0.071	0.011	**1.45E-10**	0.49	0.042	0.015	5.81E-03	6.24E-01	2.51E-01	1.09E-01
*SLC22A12*;*NRXN2*	rs10897518	C	T	0.32	0.060	0.012	1.49E-06	0.32	0.089	0.012	**9.21E-14**	0.32	0.069	0.016	2.27E-05	9.14E-01	6.66E-01	3.17E-01
*R3HDM2*;*INHBC*	rs11172134	T	A	*0*.*81*	0.049	0.015	1.17E-03	0.81	0.078	0.014	**3.78E-08**	0.80	0.069	0.019	3.23E-04	1.66E-01	4.03E-01	7.33E-01
near (5') *PDZK1*	rs1967017	C	T	0.53	-0.032	0.012	8.24E-03	0.53	-0.065	0.011	**4.89E-09**	0.53	-0.042	0.015	5.6E-03	3.86E-02	5.99E-01	2.07E-01
*GCKR*	rs780094	C	T	*0*.*59*	-0.043	0.012	2.3E-04	0.59	-0.05	0.011	5.64E-06	0.60	-0.085	0.015	**1.81E-08**	6.50E-01	2.76E-02	6.17E-02
Women				N = 9753				N = 7189				N = 4690						
*SLC2A9*	rs13129697	G	T	0.28	-0.403	0.015	**7.57E-150**	0.28	-0.412	0.018	**2.66E-111**	0.28	-0.376	0.023	**3.12E-59**	6.89E-01	3.28E-01	2.13E-01
*ABCG2*	rs2231142	G	T	0.89	-0.173	0.024	**5.56E-13**	0.89	-0.190	0.028	**2.39E-11**	0.89	-0.114	0.037	1.75E-03	6.50E-01	1.78E-01	1.02E-01
*SLC17A1*;*SLC17A4*	rs1165209	G	A	0.46	-0.096	0.014	**1.20E-11**	0.46	-0.087	0.017	1.89E-07	0.46	-0.049	0.021	2.18–02	6.99E-01	6.34E-01	1.51E-01
Men				N = 5529				N = 1058				N = 4613						
*SLC2A9*	rs16890979	C	T	0.75	0.254	0.022	**1.15E-29**	0.76	0.183	0.017	**4.47E-26**	0.76	0.176	0.026	**8.66E-12**	1.10E-02	2.25E-02	8.37E-01
	rs10805346	C	T	0.44	-0.161	0.019	**5.12E-17**	0.43	-0.170	0.015	**1.45E-31**	0.44	-0.164	0.022	**4.29E-14**	6.83E-01	9.22E-01	7.89E-01
*ABCG2*	rs2199936	G	A	0.90	-0.323	0.033	**1.06E-22**	0.89	-0.243	0.025	**1.17E-22**	0.89	-0.141	0.036	8.80E-05	5.15E-02	**1.89E-04**	1.92E-02
*SLC17A1*;*SLC17A4*	rs1165196	G	A	0.46	-0.074	0.019	1.12E-04	0.46	-0.086	0.014	**1.4E-09**	0.46	-0.056	0.021	7.96E-03	6.03E-01	5.25E-01	2.31E-01
*SLC22A12*;*NRXN2*	rs505802	C	T	0.31	0.065	0.021	1.95E-03	0.32	0.095	0.016	**1.15E-09**	0.31	0.078	0.023	7.76E-04	2.54E-01	6.76E-01	5.50E-01

^a^For the same locus, the index SNP (i.e. with the lowest P-value) may vary across stratum and when not in high LD with each other (pairwise r^2^ less than 0.5 using the SNAP proxy search tool HapMap2 rel22 data, http://www.broadinstitute.org/mpg/snap/) index SNPs are displayed separately.

A1, allele for which effect (β) is reported, A2, alternate allele, fqA1 weighted average effect-allele frequency across the combined discovery studies. Mean effect sizes (β) are inverse-variance weighted estimates; s.e. standard error of the effect estimate. Effect differences were tested using a 2-sided t test. NA: non applied as the proportion of male and female is not the same across BMI categories and the variants’ effect sizes sex-sensitive. P-value (P) reaching genome-wide significance threshold are indicated in bold. Abbreviations ov and ob stand for overweight and obese respectively.

The gene-based association test implemented in the statistical package VEGAS revealed one novel locus, *CLK4*, reaching the gene-based genome-wide significance in the obese-men stratum only (P-value = 2 x 10^-6^, just below the Bonferroni corrected gene-based threshold of 2.8 x 10^-6^). However, this effect was not reproduced in a replication set (S2 Fig.).

A complete list of top associated genes in the gene based analysis is reported in S6 Table down to the suggestive threshold for gene-based association of 10^-4^. Most encompassed known urate loci, including two of the recently reported novel urate-associated loci [2]: *A1CF*, an essential component of the apolipoprotein B mRNA editing machinery, which is suggestive in the lean-combined sex stratum and *MLXIPL*, a carbohydrate-responsive element-binding protein, in the overweight-combined sex stratum.

### Effect size variation across BMI strata for genome-wide significant effects

Some modulation of effect sizes depending on BMI status is suggested by close inspection of the most strongly associated SNPs in each stratum (Table 2). For example, a *GCKR* SNP, rs780094, reached genome-wide significance in the obese-combined-gender stratum but no SNP within that locus reached even the suggestive threshold of association (10^-5^) in the lean-combined-gender stratum despite the larger number of individuals in the latter.

**Fig 1:**
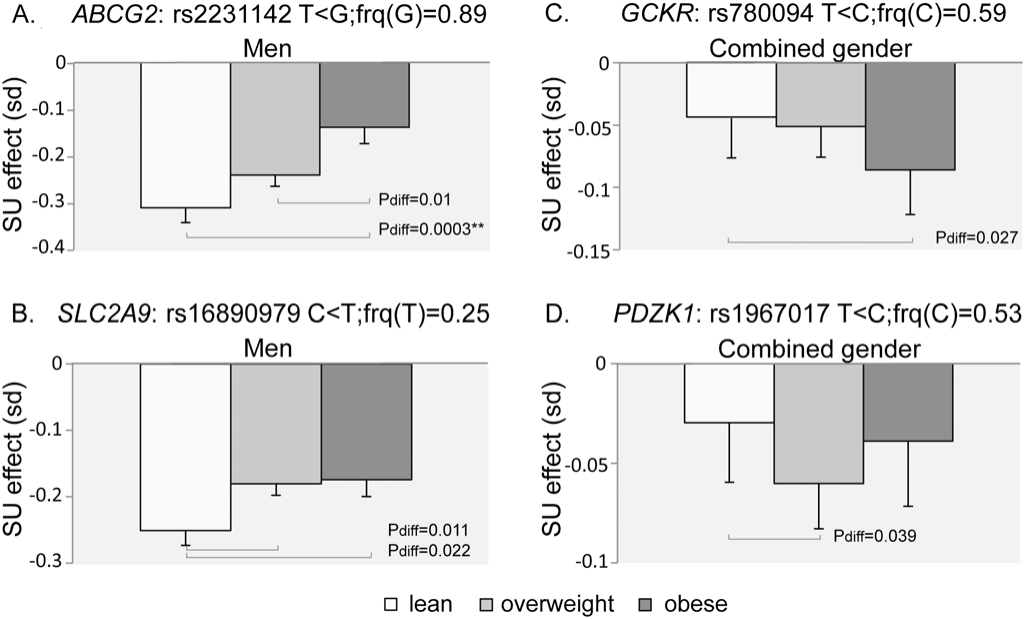
Mean effect across BMI strata of allelic substitutions at representative variants displaying genome-wide significant association with SU in at least one BMI stratum and displaying nominally significant difference in effect size across BMI strata. Effect size is on standardised age-adjusted SU levels. Error bars indicate the standard errors of the mean effect estimates within a BMI category. Horizontal lines indicate nominally significant (p < 0.05) differences in mean effect sizes between BMI categories, ** indicates significance at the 1% level taking into account the multiple comparisons performed. Differences in mean effect sizes between BMI strata were tested pairwise using the classical z-test, and P_diff_ denotes the 2-sided test corresponding P-value. Lean: BMI < 25 kg/m^2^, overweight: 25 ≤ BMI ≤ 30 kg/m^2^, obese: BMI > 30 kg/m^2^.

We formally tested the differences in SU effect sizes across BMI strata pairwise for the variants that reached the genome-wide significance threshold in at least one BMI stratum in this study (Table 2), discarding *SLC2A9* and *ABCG2* comparisons in the combined gender analysis as the proportion of male and female is not the same across BMI categories and the effect sizes of the variants are sex-sensitive. Taking a Bonferroni corrected significance threshold for the number of independent SNPs analysed in different settings (0.05/(14*3) = 0.0012), only one locus, *ABCG2*, showed a statistically significant difference in effect size between obese and lean men (Table 2) and the trend between BMI categories and effect on SU level seemed linear (Fig. 1A). The magnitude of the effect on urate for the *ABCG2* index SNP was more than halved in the obese category compared to the lean category (effect of rs2231142 allelic substitution: 95% CI 0.257 to 0.389 in lean men versus 95% CI 0.069 to 0.213 in obese male) making the magnitude of effect in obese men similar to that seen in women (95% CI 0.125 to 0.221 in lean women). SNPs at three additional loci reached nominal significance (Fig. 1B-D).

### Effect size variation across BMI strata genome-wide

**Fig 2:**
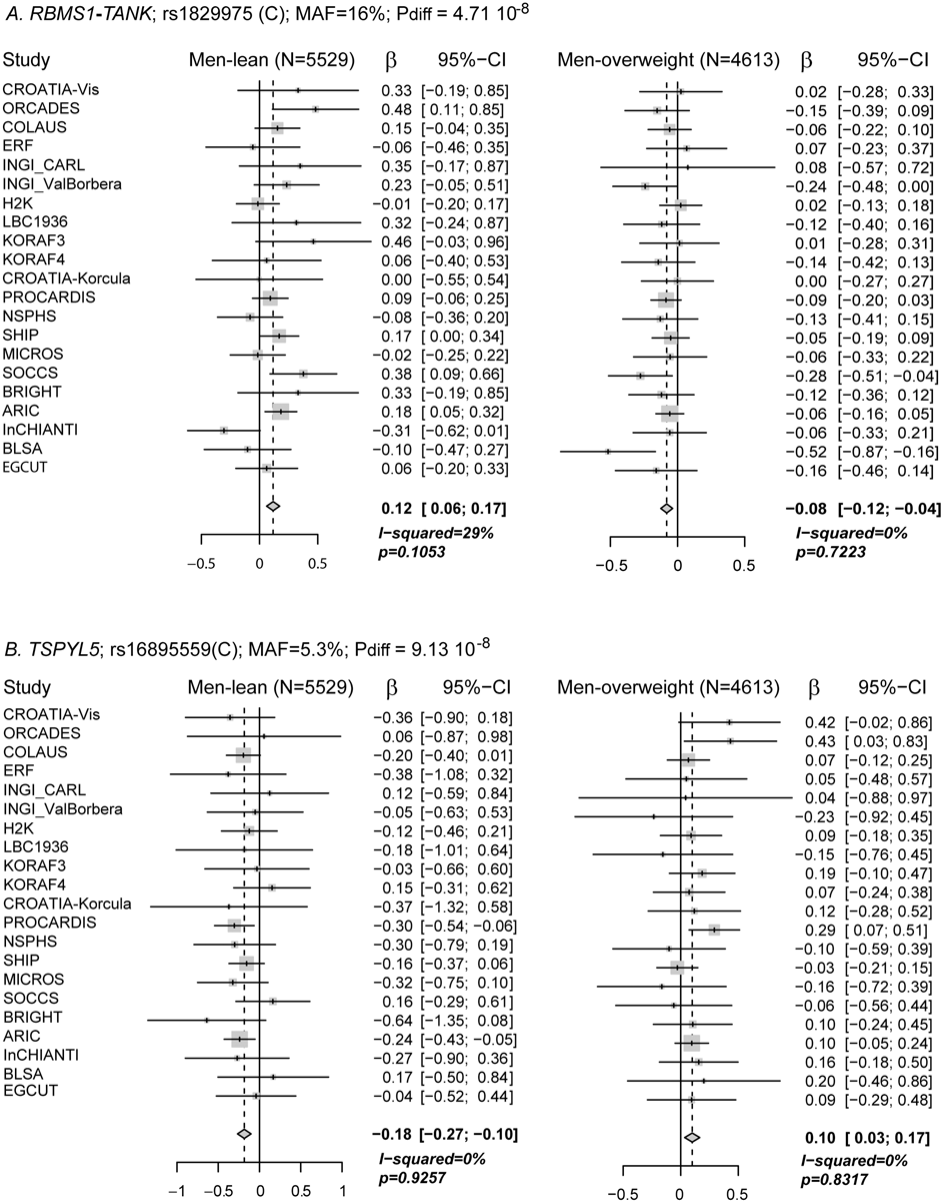
Forest plots of effect sizes within BMI stratum for variants with the two most significant mean effect size differences between BMI stratum. A. *RBMS1*-*TANK* locus and B. *TSPYL5* locus. The overall inverse—variance-weighted mean effect per BMI stratum is calculated assuming fixed effect across studies and represented by a lozenge, associated P-value displayed as P. Measure of heterogeneity between studies is reported (I-squared) with associated P-value for significance (p). P_diff_ is the test of difference in mean-effect size P-value. For study abbreviations and references, see S1 Table.

The same tests were also done genome-wide to investigate potential BMI-sensitive SNPs of opposite effect between strata. QQ plots for those analyses (S3 Fig.) showed no evidence for an excess of false positive results (genomic inflation factors ranged from 1.004 to 1.016). The most significant effect-differences (P_diff_ < 10^-5^) for all nine comparisons, after quality control for low frequency variants, are reported in S7 Table together with results for the 28 urate loci known to date, none of which reaching a P_diff_ lower than 10^-5^. The lowest P-values were from the lean-obese and lean-overweight comparisons, all in loci not previously associated with urate and displaying different direction of effects in the lean and obese/overweight strata (Fig. 2 and S4 Fig.). The variant rs1829975, intergenic in *RBMS1-TANK*, a region that has been associated with several obesity related traits [25,26,27], reached the genome-wide significance threshold (P_diff_ < 5*10^-8^) in the men lean-overweight contrast. The second most significant difference, P_diff_ = 9.13 x 10^-8^, was also in the men lean-overweight contrast for a variant 5’ of the gene *TSPYL5*, a gene coding the testis specific Y-encoded-like protein 5 that has been recently suggested to regulate estradiol produced by adipocytes [28]. The most significant loci for the lean-obese comparisons were intergenic *ARL5B-PLXDC2* and *LASS3* for the men (P_diff_, respectively, 1.1 x 10^-7^ and 2.2 x 10^-7^) and *RBFOX3* for women and combined gender (P_diff_~ 4 x 10^-7^) which had suggestive main effect in the obese women stratum.

### Interaction effect in linear regression models

To see whether a simple linear modelling of the BMI by SNP interaction (see methods) would uncover the same loci as the stratified analysis, interaction term analyses in linear models were conducted in a subset of the discovery studies. Only those with an inflation factor less than 1.2 were combined in a meta-analysis. Two common variants, one intergenic *EROL1B-EDARADD* and one in the *RBFOX3* gene, displayed P-values just below the genome wide significance for a BMI*SNP interaction in the combined-sex analysis (rs10802528 P_inter_ = 7.78 x 10^-8^ and rs898534 P_inter_ = 9 x 10^-8^, Table 3 and full list of most significant results in S8 Table). SNPs at these loci also displayed suggestive interaction in the women-only analysis. The *ERO1LB-EDARADD* locus remained suggestive in a sensitivity analysis with only the combined sex studies with the lowest genomic inflation analysed (lambda < 1.05, S9 Table), while index SNP rs898534 in *RBFOX3*’s P-value drops to 1.5 x 10^-4^. For a fair comparison, the tests for difference of main effects between BMI strata presented in S7 Table were recalculated using the exact subset of studies for which BMI*SNP term results were analysed (S9 Table) and led to similar conclusions. Noticeably, while the top loci in the lean versus obese comparisons come up as top loci in the linear fitting of an interaction term (S8 and S9 Tables), none of the loci ranking high in the lean versus overweight strata reached suggestive significance in the linear modelling despite the strongest P_diff_ P-values, suggesting a non-linear mode of action for those.

**Table 3:** Most significant BMI x SNP interaction terms for urate GWAMA.

							Discovery_Metaanalysis N = 28610				Replication_Metaanalysis N = 13959				Combined_Metaanalysis N = 42569			
Locus	SNP	A1	A2	chr	Pos(36)	fqA1	β_inter_	s.e.	P_inter_	I^2^	β_inter_	s.e.	P_inter_	I^2^	β_inter_	s.e.	P_inter_	I^2^
*ERO1LB*-*EDARADD*	rs10802528	G	T	1	234573438	0.55	0.010	0.002	7.8E-08	0%	0.003	0.003	3.4E-01	0%	0.008	0.0015	2.94E-07	0%
*RBFOX3*	rs898534	G	A	17	74785108	0.88	-0.016	0.003	9 E-08	0%	-0.009	0.005	5.2E-2	0%	-0.014	0.003	2.61E-08	0%

A1, allele for which effect (β) is reported; A2 alternate allele, fqA1 weighted average effect-allele frequency across studies meta-analyzed; s.e. standard error of the effect estimate, I^2^ meta-analysis heterogeneity statistic. The interaction term is modelled within a linear model where standardised SU levels (after adjustment for age and sex) is regressed on BMI, SNP and their interaction. βinter is the regression coefficient for the interaction term.

We attempted replication of the linear interaction seen in the combined-sex analysis in a replication set consisting of six studies. Model-robust estimates of effects’ standard errors were calculated to avoid inflated λ_GC_ statistic commonly seen when using classical regression approaches [21]. Top results for this replication set and the combined samples are reported in S9 Table. Both *RBFOX3* and *ERO1L-EDARADD* SNPs showed consistent direction of interaction effect between discovery and follow-up sets and a low level of heterogeneity across studies and *RBFOX3* index SNP reached genome-wide significance in the combined dataset (Table 3).

### Pathway analysis

We used a recently developed pathway analysis method where pathway associations are tested following circular permutations of all the GWAS SNPs P-values [22] and compare enriched KEGG defined pathways in all nine strata. Results (S10 Table) did not uncover any pathway reaching the genome-wide significance threshold defined by a strict Bonferroni correction using 229 pathways and nine analyses (P = 2.43 x 10^-5^) but this threshold is very conservative given that many pathways are interconnected or overlapping and the combined and sex separate analyses are not independent. The most significant pathways were the ribosome pathway (P = 3 x 10^-4^) in overweight women, glycosaminoglycan degradation in obese men (P = 6 x 10^-4^) and N-glycan biosynthesis in lean women (P = 6 x 10^-4^).

N-glycan biosynthesis (KEGG pathway hsa00510- N = 43 genes) is particularly compelling as its ranking amongst associated pathways is stable through the variable sample sized analyses for the same BMI stratum: it ranks top in all the lean meta-analyses (rank = 1 in combined-gender and women, rank = 21 in men), while it is medium-ranked in all the overweight analyses (rank = 68 in combined-gender, rank = 69 in women and rank = 103 in men) and amongst the lowest ranks in all the obese strata (rank = 217 in combined-gender, rank = 223 women and rank = 218 men). The list of genes out of the 43 genes in this pathway with at least one SNP nominally significantly associated with urate levels (P <0.05) in either BMI stratum in the combined-gender analyses are listed in S11 Table.

## Discussion

No novel locus with a genome-wide significant main effect on SU was uncovered when performing GWAS within the three BMI strata investigated, suggesting that changes in BMI do not switch on a yet unknown major urate locus. However, many loci reached suggestive level of SU association in a BMI dependent fashion and/or displayed suggestive difference in main effects across BMI categories that may collectively account for a substantial amount of BMI-sensitive SU variation.

One weakness of this study is its relatively modest size. Gene by environment (GxE) detection requires a larger sample-size than that required for the detection of main effects of comparable magnitude [29] (a rule of thumb proposed for case control design is a four time larger study [30]). Data from over 200,000 individuals were required to confirm the attenuation of FTO obesity risk genotype by physical activity [31] with the reported interaction term significant, P_inter_ = 0.001, because only one candidate gene was tested. Few scans for GxE interaction have been performed genome-wide to date [32–37]. A stratification strategy was used to uncover novel women-specific genetic effects in waist-related phenotypes with strong statistical support using a very large dataset [36] and gave support for stronger effects of the known to date type 2 diabetes genetic risks variants in lean compared to obese individuals [35]. Other studies have reported modest (P_inter_ at best 10^-4^) interaction effect after testing for a joint effect of the main SNP effect and interaction term with the significant results driven by the main SNP effect [32,34]. Joint effect meta-analysis (JMA) was implemented fairly recently [38] and best suited when both main and interaction effects are present.

Our study was additionally challenged by using a crude readout, BMI, where similar measures can reflect very different physiological status, e.g high BMI could correspond to high visceral fat deposition as well as low visceral fat deposition but high muscle mass. It would certainly benefit from more specific measures of environmental exposures for example, of diet (fructose, fat content, alcohol intake) or amount of physical activity or of metabolic status of the subject.

Despite these limitations, this is the largest investigation of the interplay between genetic variants influencing urate and BMI status to date and it provides novel, biologically supported, hypotheses that warrant further investigations.

Of the known urate loci, there was weak statistical evidence for modulation of *SLC2A9* variant effects by BMI and no support from previous reports [9,16] of a consistent BMI modulating effect. By contrast, statistically significant change was observed for *ABCG2* in men, with a fan-shaped interaction pattern and diminution (by half) of the genetic variant effect size in obese compared to lean men on average. The ATP-binding cassette transporter ABCG2 has been established as a high capacity urate transporter, is expressed in renal proximal tubules, liver and intestines, and the hyperuricemia causal Q141K mutation has been shown to reduce urate transport rates [39]. Surrounding lipids, ATP concentrations, cholesterol and bile acids have been shown to modulate activity of ABCG2 in vitro [40]. Interestingly, BMI-dependent effects of Q141K on urate response to acute fructose exposure have been recently reported [41]. A stronger effect of *GCKR* variant in the obese strata was only suggestive but it is well supported by the equivalent doubling in the lowering effect reported for the *GCKR* pleiotropic rs780094 T allele for fasting insulin and glucose in high-BMI participants compared to low-BMI participants, supplementary Table 2 of [32]. It is also consistent with the finding that adjustment for triglyceride (TG) level as potential mediator/confounder attenuates *GCKR* rs780094 variant urate association [10].

*RBFOX3* and *EROL1B* were the top loci showing interaction with BMI status using linear models (with *RBFOX3* index SNP reaching genome-wide significance in the combined discovery and look-up studies GWAMA). Both loci displayed the strongest evidence of a significant difference in SNP main effect when the lean and obese stratified samples were compared (S7 Table), analyses in which no individual study showed a high inflation factor or high heterogeneity across studies, supporting genuine interaction with BMI and in a linear fashion. We noted that, in contrast to those, the two top ranking loci from the stratified analyses comparisons (both for men lean-overweight contrasts) were not significantly interacting with BMI when using a linear model of interaction, and would require replication using the same methodology to be confirmed. *RBFOX3* is a neuronal nuclear marker expressed in the Arcuate nucleus in the hypothalamus where orexigenic and anorexigenic neurons reside. Its paralog, *RBFOX1*, has been proposed as an obesity gene [42]. *RBFOX3* (aka *HRNBP3*) was also selected together with 38 other genes in a gene-centric joint test for significant association with HDL-Cholesterol levels in a dataset combining expression data and GWAS data from independent sources [43]. A metabolic outcome of *RBFOX3* knockout in mice (international mouse phenotyping consortium) is decreased circulating alkaline phosphatase, human levels of which correlates with BMI [44] and metabolic syndrome [45], a component of which is hyperuricemia. *EROL1B* encoding for the endoplasmic reticulum oxidoreductin 1LB catalizes the formation of disulfite-bonds in the ER. It represents another good candidate for BMI interaction as it is responsive to the unfolded protein response, a signal triggered by ER stress, levels of which are elevated in state of over-nutrition [46]. ER stress response itself may induce inflammation [47] and has been correlated with increased levels of inflammation marker molecules CRP and IL6 which were both positively correlated with urate levels [48].

The “N-glycan biosynthesis” pathway acting to influence urate levels differentially in lean individuals compared to overweight or obese individuals is intriguing. One of the newly identified urate loci [2], *B3GNT4*, also acts in a complex capping reaction, of Type II Lactosamine for example, establishing a precedent for a link between glycosylation enzyme variation and urate levels. The glycolysis intermediate Fructose 6P is the main precursor of amino sugar, combining with glutamine to form glucosamine-6-phosphate. Dependence on glutamine for both purine and glycoaminoglycan biosynthesis as illustrated by the inhibition of either pathway by the glutamine analogue antagonist DON [49] also interconnects these pathways.

These links would be important to study further as glucosamine can be prescribed to patients with gout to reduce pain and inflammation but the possibility that it might influence the urate level has not been explored.

Significant changes in N-glycosylation profiles with BMI have been well documented [50,51,52]. Fitting with the urate-association results (S11 Table), core fucosylation (driven by FUT8) was noted to decrease with BMI [52] and transcript levels for the sialyltransferase gene *ST6GALT2* to be highly stimulated by the pro-inflammatory cytokines IL6 and IL8 [53] that are potentially elevated in the systemic low-grade inflammation that characterises obesity [54]. It is possible that in obese individuals flux towards O-GlcNacylation rather than towards N-glycan biosynthesis is more prominent, possibly following ER stress. O-GlcNacylation has been proposed as a nutrient sensor activated by glucose availability and correlates with insulin resistance, a common hallmark of obesity [55].

Metabolic pathways are highly inter-connected and their dys-regulation underlies many diseases. Accounting for body mass index in analyses provides a tool to link pathways to both obesity and urate homeostasis.

## Supporting Information

S1 Fig Scatter plots of BMI and serum urate in men and women from two populations used in this study. A CROATIA-Vis and B.ORCADES. Residuals from a mixed linear model adjusting serum urate (SU) levels for age and accounting for relatedness are plotted against each other. As noted in [11] the linear fit is stronger amongst women.
(TIF)

S2 Fig Forest plots for rs7711186 *CLK4* variant effect size in the male and female obese stratum in replication datasets together with those of a *SLC2A9* variant as positive control. In the discovery dataset, rs7711186 (C allele) was suggestively associated with urate in the men-obese stratum, differentially (overall effect size = 0.21, se = 0.04). Look-up in a small Polynesian study (NZL-Poly) where obesity is prominent is added under the overall meta-analysis value for the replication studies, all of European ancestry (represented by lozenge). *For this Polynesian study only the *SLC2A9* variant rs11942223, in LD (r^2^ = 0.6) with variant rs13129697, was available and used in the figure.
(TIF)

S3 Fig QQ plots for difference in SU effect statistics in all nine comparisons performed: lean versus overweight, lean versus obese and overweight versus obese in combined-gender (ALL) or sex-stratified (MEN, WOMEN) samples. The ordered observed squared t statistic are plotted against the ordered expected statitics of the null, chi2, distribution, where t = (β_bmicat1_ - β_bmicat2_)/sqrt(SE_bmicat1_^2^ + SE_bmicat2_^2^-2r(SE _bmicat1,_ SE _bmicat2_)),with β_bmicat_ and SE_bmicat_ the meta-analysis weighted beta-estimates and their corresponding standard errors and r the Spearman rank correlation coefficient between meta-analyzed beta-estimates in the BMI categories compared across all SNPs. Inflation coefficients, λ_GC,_ are reported for each plot in the left upper corner.
(TIF)

S4 Fig Forest plots of effect sizes within BMI stratum for variants showing the most significant mean effect size differences (associated P-value, P_diff_) between BMI stratum genome-wide, in the combined-gender (all) strata. The overall inverse—variance-weighted mean effect per BMI stratum is calculated assuming fixed effect across studies and represented by a lozenge, associated P-value displayed as P. Measure of heterogeneity between studies is reported (I-squared) with associated P-value for significance (p). For study abbreviations and references, see S1 Table.
(TIF)

S1 Table Study description for each study site. (DOC)

S2 Table Individual study summary statistics for serum urate levels (SU) within the nine BMI/gender categories analysed. SU unit is in mg/dl, sd stands for standard deviation, N is the number of individuals with BMI and SU measures.
(XLS)

S3 Table Study-specific genotyping, imputation information and analysis softwares. (XLS)

S4 Table List of inflation factors (λ) for each sub-analysis at individual study level. Inflation factors were calculated after filtering out poorly imputed variants and low frequency variants (MAF < 1% for main effect analysis in BMI-stratified GWAS (λ*), MAF < 5% for SNP*BMI interaction term analysis (λ**). NA flags analysis not performed. *** indicates that model-robust regression method was used.
(XLS)

S5 Table List of loci encompassing SNP(s) with SU association suggestive P-value (5 x 10^-8^ = <P <10^-5^) in the nine stratified GWAMA performed. Only the information pertaining to the SNP with the lowest P-value (index SNP) is listed. Lower allele frequency variants (1%<MAF <5%) are reported if the meta-analysis included at least four populations and if the contribution of any single study, as calculated by the meta R package, was lower than 30%. A1, allele for which effect (β) is reported; A2 alternate allele, frq(A1) weighted average effect-allele frequency across studies. Associations reported in the vicinity of the urate index SNP (in a 150kb region centred on the SNP) in the NHGRI GWAS catalogue (29_10_2013 update) are listed; highlighted red, the ones with same index SNP or index SNP in high to moderate linkage disequilibrium (r2 >0.4).
(XLS)

S6 Table List of significant and suggestive loci (P-value < 10^-4^) from the nine BMI stratified GWAMA in the gene-based association test implemented in VEGAS. Novel loci are shaded in grey. In bold, gene reaching genome-wide significant association with serum urate levels (P < 2.10^-6^).
(XLS)

S7 Table List of loci with SNP(s) displaying the strongest evidence of SU mean effect size difference across BMI strata in the discovery studies. Effect differences were tested using a t test. All loci with SNP displaying a P_diff_ < 10^-5^ are listed with representative index SNP of lowest P-value (the total number of SNPs with suggestive P-value per loci is listed in N suggestivSNPs column). Low MAF SNPs were filtered as in S5 Table. Additionally, Pdiff values for the 28 known urate loci [2] are listed with index SNP from the published data. Locus in bold indicates that the difference in effect size between BMI strata reached genome-wide significance. P-value in bold for the known urate loci are those reaching the nominal threshold of 0.05. Locus with asterix had index SNP with main effect reaching suggestive level of association (P < 10^-5^) in the BMI stratified urate GWAMA analysis (S5 Table).
(XLS)

S8 Table List of loci with suggestive (P_inter_ < 10^-5^) SNPxBMI interaction term using regression based method. Studies with inflation factor greater than 1.2 were not included in the analysis. For the combined-gender analysis, the CoLaus study was analysed as a replication study to balance discovery and replication sets. SNP with low MAF (< 5%) were excluded prior to meta-analysis. Results for the discovery, replication and combined sets are presented. Locus in bold indicates a genome-wide significant interaction effect. Shaded are loci common with S9 Table (list of loci with suggestive difference in urate main effects between BMI stratified GWAMA)
(XLS)

S9 Table Results obtained as in S7 Table when analysis is restricted to the subset of studies (N = 16) used for BMI by SNP interaction testing using a regression-based method and with markers of MAF > 5% for direct comparison. Shaded are loci displaying suggestive association in linear interaction model(listed in S8 Table).
(XLS)

S10 Table Results from the Pathway analysis tool implemented in the genomicper R package in the nine stratified urate GWAMA performed. (XLS)

S11 Table List of genes in the KEGGs N-glycan biosynthesis pathway, hsa00510, harbouring at least one SNP with a serum urate GWAMA P-value (P) nominally significant in one of the three combined-gender BMI categories analysed. N-glycan biosynthesis step coded 1 = N-glycan lipid-linked oligosaccharide precursor synthesis 2 = high mannose oligosaccharide to an Asparagine residue transfer and N-glycan trimming and branching 3 = more elaborate capping reactions
(XLS)
