# Supplementary figures and images for: Modulation of Genetic Associations with Serum Urate Levels by Body-Mass-Index in Humans
Source: PLoS One. 2015 Mar 26;10(3):e0119752. doi: 10.1371/journal.pone.0119752 (PMC4374966; doi:10.1371/journal.pone.0119752)

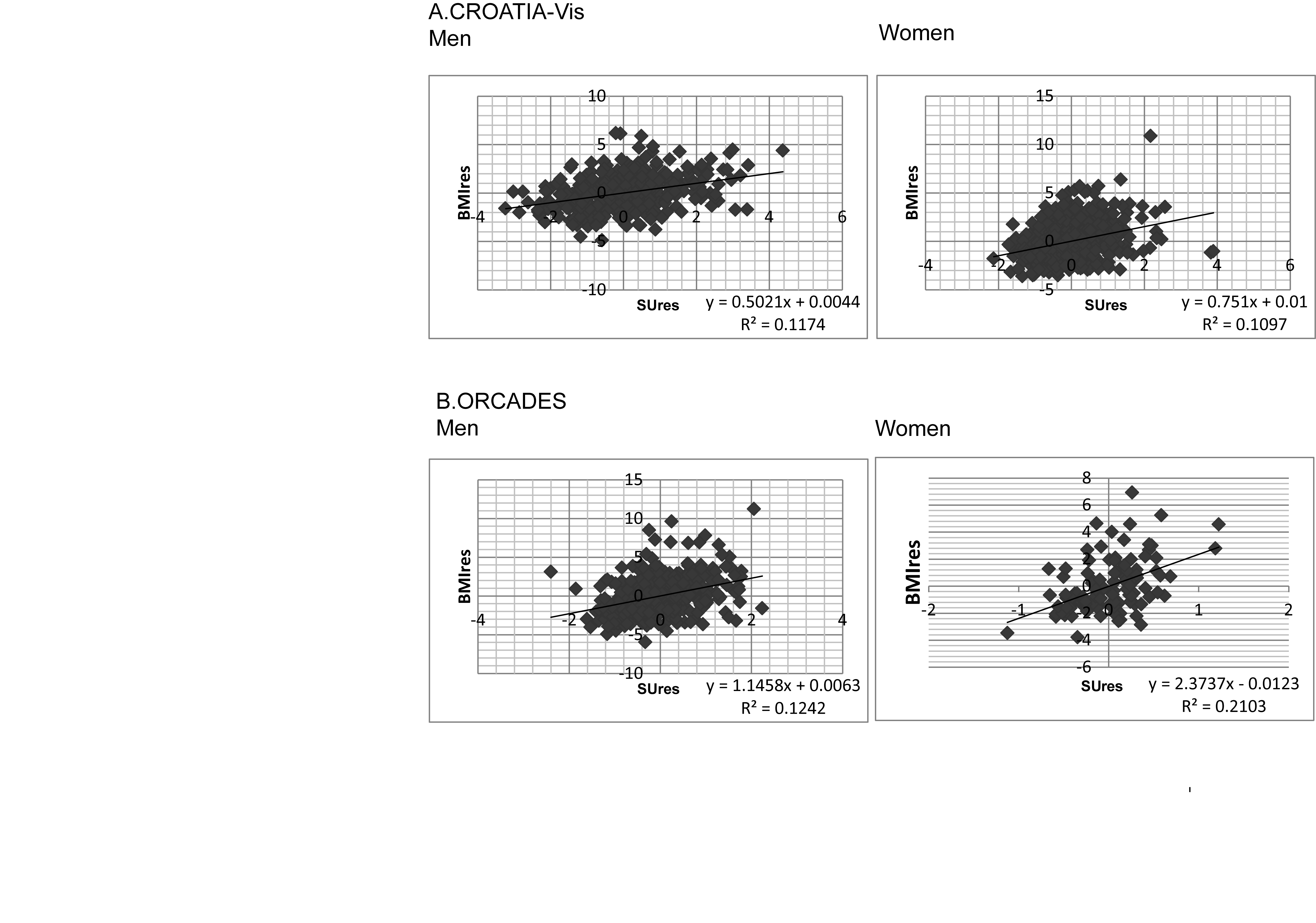

Supplement: S1 Fig — A CROATIA-Vis and B.ORCADES. Residuals from a mixed linear model adjusting serum urate (SU) levels for age and accounting for relatedness are plotted against each other. As noted in [11] the linear fit is stronger amongst women. (TIF) [file pone.0119752.s001.tif]

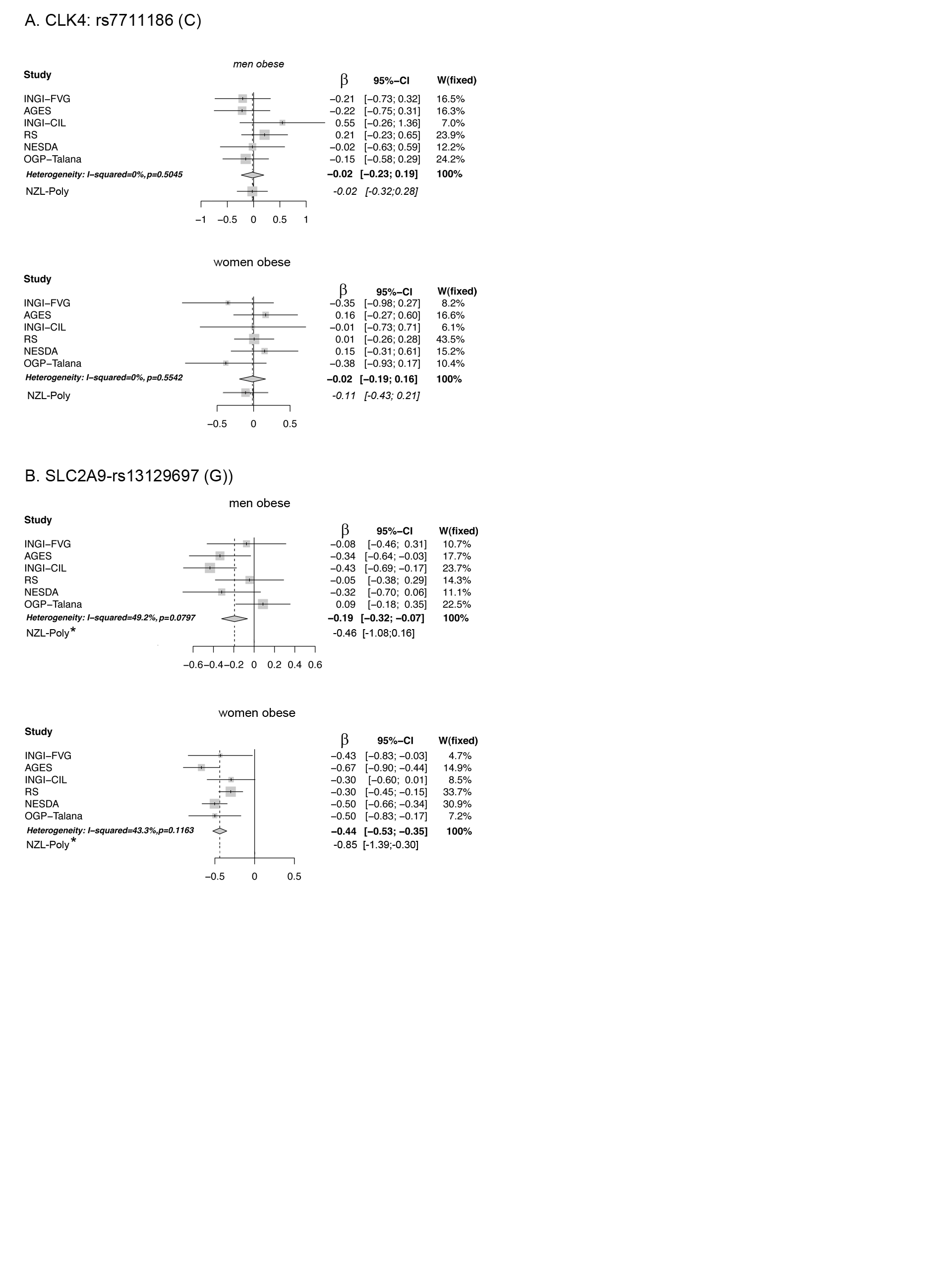

Supplement: S2 Fig — In the discovery dataset, rs7711186 (C allele) was suggestively associated with urate in the men-obese stratum, differentially (overall effect size = 0.21, se = 0.04). Look-up in a small Polynesian study (NZL-Poly) where obesity is prominent is added under the overall meta-analysis value for the replication studies, all of European ancestry (represented by lozenge). *For this Polynesian study only the SLC2A9 variant rs11942223, in LD (r2 = 0.6) with variant rs13129697, was available and used in the figure. (TIF) [file pone.0119752.s002.tif]

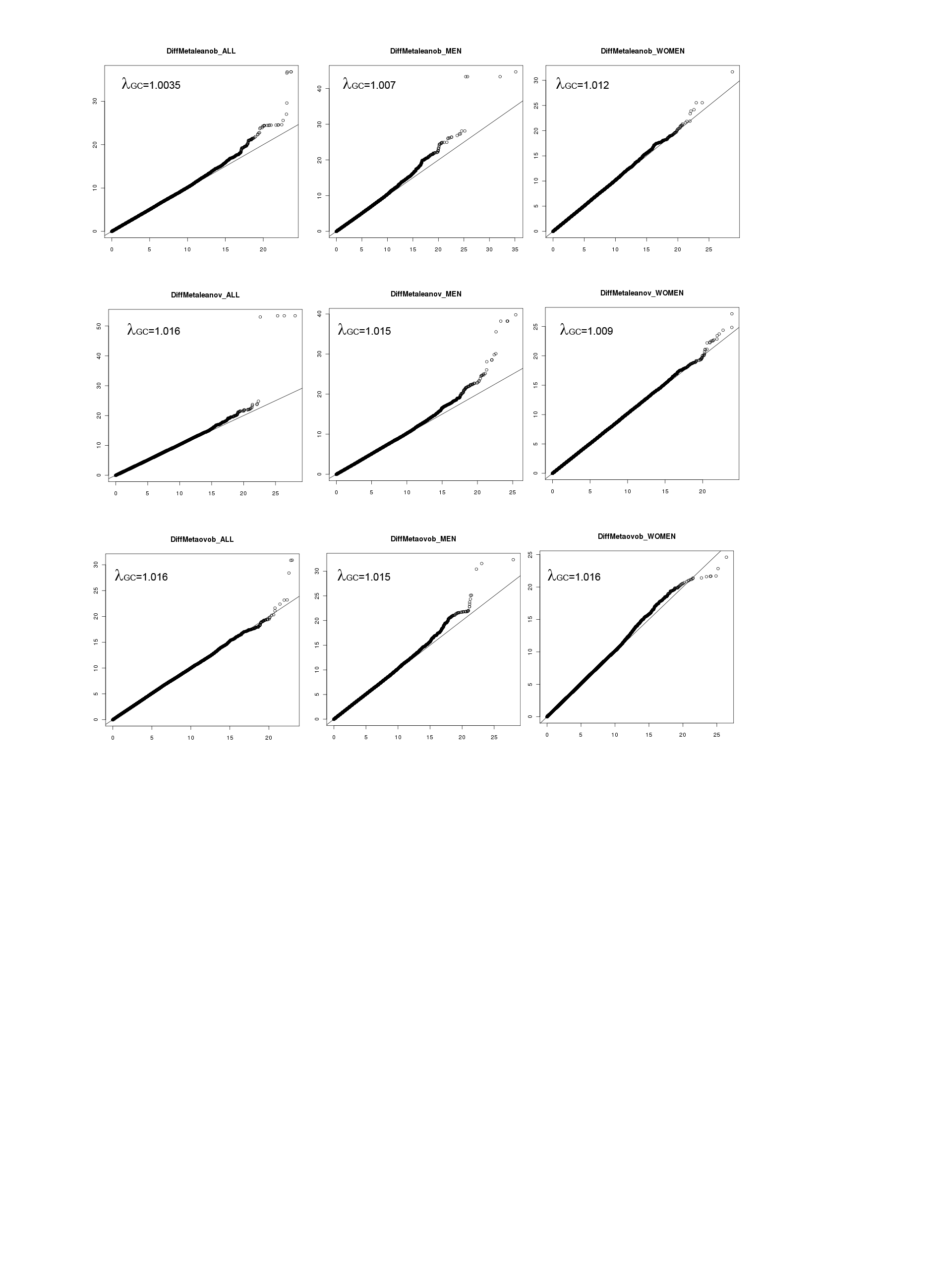

Supplement: S3 Fig — The ordered observed squared t statistic are plotted against the ordered expected statitics of the null, chi2, distribution, where t = (βbmicat1 - βbmicat2)/sqrt(SEbmicat1 2 + SEbmicat2 2-2r(SE bmicat1, SE bmicat2)),with βbmicat and SEbmicat the meta-analysis weighted beta-estimates and their corresponding standard errors and r the Spearman rank correlation coefficient between meta-analyzed beta-estimates in the BMI categories compared across all SNPs. Inflation coefficients, λGC, are reported for each plot in the left upper corner. (TIF) [file pone.0119752.s003.tif]

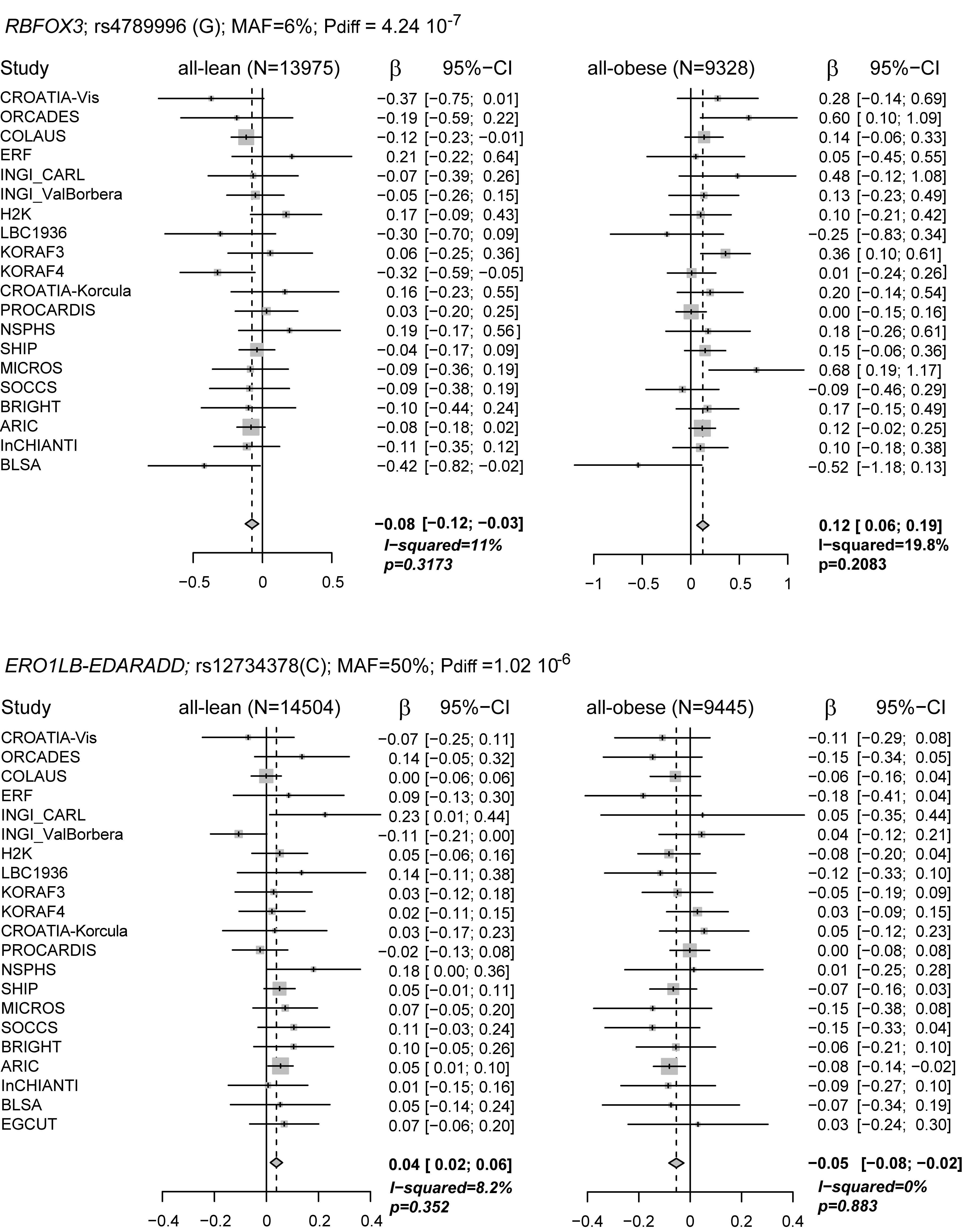

Supplement: S4 Fig — The overall inverse—variance-weighted mean effect per BMI stratum is calculated assuming fixed effect across studies and represented by a lozenge, associated P-value displayed as P. Measure of heterogeneity between studies is reported (I-squared) with associated P-value for significance (p). For study abbreviations and references, see S1 Table. (TIF) [file pone.0119752.s004.tif]
