# Supplementary material for: Modulation of Genetic Associations with Serum Urate Levels by Body-Mass-Index in Humans
Source: PLoS One. 2015 Mar 26;10(3):e0119752. doi: 10.1371/journal.pone.0119752 (PMC4374966; doi:10.1371/journal.pone.0119752)
Supplement: S1 Table — (DOC) [file pone.0119752.s005.doc]

| **Study Name** | **Ethics authorities** | **Study Design** | **Total Genotyped Sample Size** | **Exclusion Criteria for Study Participation or Disease Enrichment** | **Exclusions for present study** | **Population Stratification** | **UA Measurement and QC** |
| --- | --- | --- | --- | --- | --- | --- | --- |
| Discovery |  |  |  |  |  |  |  |
| Atherosclerosis Risk in Communities (ARIC) Study | Ethics boards of all participating sites (Forsyth County, NC; Jackson, MS; the northwestern suburbs of Minneapolis, MN; and Washington county, MD) | Prospective, population-based | 9,713 of European ancestry | none | Of the 9713 genotyped individuals of European ancestry, we excluded 658 individuals based on discrepancies with previous genotypes, disagreement between reported and genotypic sex, one randomly selected member of a pair of first-degree relatives, or outlier based on measures of average DST or more than 8 SD away on any of the first 10 principal components. | Two principal components were associated with uric acid measurements and included as covariates in the regression. | UA was measured using the uricase method at study visit 1. Repeated measurements of UA in 40 individuals, taken at least one week apart, yielded a reliability coefficient of 0.91, and the coefficient of variation was 7.2%. |
| Baltimore Longitudinal Study of Aging (BLSA) | Ethics authorities of the National Institute on Aging of US NIH | Prospective, population-based | 1,230 subjects | none | Of the 1230 genotyped subjects, genetic relatedness was assessed using PCA analysis using the HapMap population as reference. Out of the 857 subjects of European ancestry, 5 subjects were excluded for low genotyping (< 98.5%), 4 subjects were removed for sex misspecification. From the 848 subjects with European ancestry passing quality control, 718 subjects with uric acid data was used for this study. | Use top two principle components included as covariates in the regression model. | UA was measured using the uricase method (Johnson and Johnson, VITROS chemistry system). |
| British Genetics of Hypertension (BRIGHT) | Multi-Centre and Local Research ethics committees | hypertensive participants and normotensive controls | 1,743 of European ancestry | none | Individuals with call rates below 0.97 were excluded. | none | UA concentrations were measured using an  uricase method (Hitachi, Roche Diagnostic). |
| Cohorte Lausannoise (CoLaus) Study | Ethics committee of the canton Vaud | population based | 5,636 of European ancestry | none | Individuals with call rate below 90% were excluded. The younger of 1^st^/2^nd^ degree related pairs were removed from the analysis. | First two ancestry principal components were used as covariates. | Serum uric acid was measured by uricase-PAP (1.0% - 0.5% maximum inter and intra-batch coefficients of variation). |
| CROATIA-Korcula | the Medical School, University of Zagreb (2001; ref 018057 and 2006; ref 04-1097-2006), the Multi-Centre Research Ethics Committee of the National Health Service UK (2001; MREC 01/0/71), and NHS Lothian (2011; REF 11/AL/0222) | cross-sectional, population-based | 971 | none | 898 individuals left after QC based on genotyping quality, sex and ancestry check | None of the first 3 principal components strongly associated with uric acid; relatedness of participants taken into account using a mixed linear model with the polygenic effect set as random effect. | UA was measured using the uricase UV photometry method in "Labor Centar“ biochemical lab, Bukovcev trg 3, 10000 Zagreb Croatia (www.laborcentar.hr). |
| CROATIA-Vis | the Medical School, University of Zagreb (2001; ref 018057 and 2006; ref 04-1097-2006), the Multi-Centre Research Ethics Committee of the National Health Service UK (2001; MREC 01/0/71), and NHS Lothian (2011; REF 11/AL/0222) | cross-sectional, population-based | 991 | none | 924 individuals left after QC based on genotyping quality, sex and ancestry check | None of the first 3 principal components strongly associated with uric acid; relatedness of participants taken into account using a mixed linear model with the polygenic effect set as random effect. | UA was measured using the uricase UV photometry method in "Labor Centar“ biochemical lab, Bukovcev trg 3, 10000 Zagreb Croatia (www.laborcentar.hr). A subset of 774 samples had also been measured independently in the Institute for Clinical Chemistry and Laboratory Medicine, University Hospital Regensburg, Germany. Pearson correlation between the two urate measurements was 94% . |
| Estonian Genome Center of University of Tartu (EGCUT) | Ethics Review Committee on Human Research at the University of Tartu | Prospective, population-based | 931 of European ancestry | none | Low genotyping quality (call rate <98%, MAF <1%, HWE p-value 10E-6); disagreement between reported and genotypic sex, one randomly selected member of a pair of first-degree relatives | Three principal components were associated with uric acid measurements and included as covariates in the regression. | UA was measured using the uricase method |
| Erasmus Ruchphen Family (ERF) Study | Medical Ethics Committee of the Erasmus University | Family based | 2,385 | none | none | Score test for association in related people implemented in R package GenABEL was used to control for family related ness. | UA concentrations were measured using an uricase/peroxidase method (DVIA1650-Autoanalyzer, Siemens Healthcare Diagnostics) |
| Health 2000  (H2K) | Ethics Committee for Epidemiology and Public Health of the Hospital district of Helsinki and Uusimaa, Finland. | Population-based | 2,123 Finns | none | Samples with discrepancy between reported and genotypic sex were excluded. For pairs with pi_hat > 0.2 one of the pairs was excluded. Individuals with 0.05 < pi_hat < 0.2 to many other individuals were excluded. | NA | Uricase method, a colorimetric enzymatic method (Thermo Fisher Scientific, Vantaa, Helsinki). |
| Invecchiare in Chianti (InCHIANTI) study | Ethics Committee of the Italian National Research Council of Aging “I Fraticini”, Florence,Italy | Prospective, population-based | 1,230 European ancestry | none | Of the 1231 genotyped subjects, 22 subjects were removed based on genotyping completeness (<97%), low heterozygosity (<0.3), or sex misspecification. 1205 subjects with uric acid data was used for the analysis. | Genomic Control | Plasma UA (mg/dl) was measured using an enzymatic-colorimetric method (Roche Diagnostics, GmbH, Germany). The lower limits of detection were 0.2 mg/dl, range 0.2–25.0 mg/dl, intra- assay and inter assay coefficients of variation (CV) were 0.5 and 1.7%, respectively. |
| INGI-Carlantino  (INGI_CARL) | Ethics Committee of the Burla Garofolo children Hospital in Trieste**.** | Population-Based | 659 | none | Removed people with call rate <0.95  or too high IBS or heterozigosity.  Removed people that did not pass sex chromosome checks or were < 18 years of age. | Corrected using mixed model regression analysis. | UA was measured with the colorimetric method using Targa 3000 from Biotecnica Instruments |
| INGI-Val Borbera | San Raffaele Hospital and Regione Piemonte ethics committtees | Family Population-based | 1,665 | none | Of the 1665 participants who underwent genotyping, we made the following exclusions: sample call rate <95% (n=1) | Score test for association in related people implemented in GenABEL was used to control for family related ness. | UA was measured using HITACHI 917 ROCHE and Unicel Dx-C 800 BECKMAN |
| KORA F3 | The KORA Augsburg studies were approved by the local Ethics Committee | population-based | 1,644 | none | Only subjects with overall genotyping efficiencies of at least 93% were included. In addition the called gender had to agree with the gender in the KORA study database. | none | Non-fasting blood samples were obtained from study participants. UA analyses were carried out on fresh samples. UA concentrations were measured using an uricase method (URCA Flex, Dade Behring). |
| KORA F4 | The KORA Augsburg studies were approved by the local Ethics Committee | population-based | 1,814 | none | Only subjects with overall genotyping efficiencies of at least 93% were included. In addition the called gender had to agree with the gender in the KORA study database. | none | Fasting blood samples were obtained from study participants. UA analyses were carried out on fresh samples. UA concentrations were measured using an uricase method (URCA Flex, Dade Behring). |
| Lothian Birth Cohort 1936 (LBC1936) | The Multi-Centre Research Ethics Committee for Scotland (MREC/01/0/56) and from Lothian Local Research Ethics Committee (LREC/2003/2/29). | Retrospective and prospective community-based cohort study (1) | 1,005 of European ancestry | none | Individuals with a disagreement between genetic and reported gender were removed (n = 12 in LBC1936). Relatedness between subjects was investigated and, for any related pair of individuals, one was removed [PI_HAT (proportion of IBD) > 0.25, n = 8 in LBC1936). Samples with a call rate ≤ 0.95 (n = 16 in LBC1936), and those showing evidence of non-European descent by multidimensional scaling, were also removed (n = 1 in LBC1936). | None of the four extracted principal components were associated with uric acid measurements so were not included in the model. | Serum uric acid was determined using the VITROS URIC DT slide method performed using the VITROS URIC DT slide and the VITROS Chemistry products DT Calibrator Kit on VITROS DT60/DT60 II Chemistry systems (VITROS). This was performed at the Combined Biochemistry and Haematology Labs, Western General Hospital. |
| MICROS | Ethics Committee of the Autonomous Province of Bolzano | cross-sectional, population-based | 1,345 | none | 1,268 individuals left after QC based on genotyping quality, sex and ancestry check. | None of the first 3 principal components strongly associated with uric acid but village of origin kept as cofactor; relatedness of participants taken into account using a mixed linear model with the polygenic effect set as random effect. | UA was measured using the uricase /peroxidase method. |
| NSPHS | Regional ethics committee at the University of Uppsala (Regionala Etikprovningsnamnden, Uppsala,Dnr 2005:325). | cross-sectional, population-based | 700 | none | 656 individuals left after QC based on genotyping quality, sex and ancestry check | None of the first 3 principal components strongly associated with uric acid; relatedness of participants taken into account using a mixed linear model with the polygenic effect set as random effect. | UA was measured using the uricase /peroxidase method. |
| The Orkney Complex Disease Study  (ORCADES) | The Orkney and North of Scotland Local Research Ethics Committees. | cross-sectional, population-based | 920 | of non-orcadian ancestry | 889 individuals left after QC based on genotyping quality, sex and ancestry check. | None of the first 3 principal components strongly associated with uric acid; relatedness of participants taken into account using a mixed linear model with the polygenic effect set as random effect. | UA was measured using the uricase /peroxidase method in the Balfour Hospital, Kirkwall, UK. A subset of 718 samples had also been measured independently in the Institute for Clinical Chemistry and Laboratory Medicine, University Hospital Regensburg, Germany. Pearson correlation between the two urate measurements was 99% . |
| PROCARDIS | Ethics Committees of the participating institutions (<http://www.procardis.org/>) | Case-Control study of CAD | 3,742 | none | Dataset was prefiltered for individuals with success rate <95%, ancestory outliers on PCA, heterozygosity, IBC | Country of Origin was added as a covariate, population stratification was checked using PCA but was not adjusted for beyond Country of Origin. | Measured using uricase method in hospital clinical lab |
| Study of Health in Pomerania (SHIP) | Ethics Committee of the University of Greifswald. | population-based | 4,081 of European ancestry | none | 24 individuals identified as duplicated or with reported/genotyped gender mismatch | none | Uricase method, a colorimetric enzymatic method (Uric acid PAP, Boehringer) from non-fasting, fresh serum |
| SOCCS | MutiCentre Research Ethics committee for Scotand, 18 Local research ethics committees, 18 Caldicott guardians and 16 NHS Trust management committees. | colorectal cancer case control study, population-based | 2,024 | none | 1,984 individuals after QC, 1,105 of whom had uric acid phenotypes. | No PCs of ancestry included in analysis | UA was measured using the uricase /peroxidase method. |
| TwinsUK | The Guy’s and St. Thomas’ Hospital Local Research Ethics Committee | Twins | 5,654 of European ancestry | none | Samples exclusion criteria: (i) sample call rate < 98%; (ii) heterozygosity across all SNPs > 2 s.d. from the sample mean;(iii) evidence of non-european ancestry as assessed by PCA comparison with HapMap3 populations;(iv) observed pairwise IBD probabilities suggestive of sample identity errors;  We corrected misclassified monozygotic and dizygotic twins based on IBD probabilities. | Estimation of kinship matrix to take account of relatedness | Ektachem/Vitros system, Johnson & Johnson ClinicalDiagnostics |
| Follow-up |  |  |  |  |  |  |  |
| INGI_CILENTO | Ethics Committee of Azienda Sanitaria Locale Napoli | Population-Based study with pedigree information | 859 | none | Of the 859 participants who underwent genotyping, none was excluded | none | UA was measured using an enzymatic method. |
| Rotterdam | Medical Ethics Committee of the Erasmus University | Prospective, population based | 5,974 | none |  | none | Serum urate was measured at the baseline visit using a Kone Diagnostica reagent kit and autoanalyzer. |
| AGES Reykjavik Study | Ethics authorities of the Icelandic Heart Association and the National Institute on Aging of US NIH.GWAS was approved by the National Bioethics Committee (VSN:00-063) and the Data Protection Authority | Prospective population-based | 3,219 of European ancestry | none | none | All individuals from Iceland, with no significant stratification within the population. | Serum urate was measured at the Icelandic Heart Association using the Roche-Hitachi P-Module instrument with Roche uricase method. The coefficient of variation for the urate assay was 4.3%. |
| Netherlands Study of Depression and Anxiety (NESDA) | The Ethical Review Board of the VU University Medical Centre and subsequently by local review boards of each participating centre. | Longitudinal cohort study of individuals with depressive and/or anxiety disorder | 1,862 of western-European ancestry | Individuals were almost all cases with major depression or anxiety disorder (n=1705) | Ethnic outliers, XO and XXY samples, and samples with a call rate <95%, high genome-wide homo- or heterozygosity, excess IBS were excluded | none | UA was measured by enzymatic colorimetric test (uricase method, Roche Modular system). The coefficients of variation, over the complete measurement period, were 1.6% at a level of 0.25 mmol/l and 1.2% at a level of 0.55 mmol/l. |
| INGI-FVG | Ethics Committee of the Burla Garofolo children Hospital in Trieste. | Population-Based | 1,471 | none | Removed people with call rate <0.95 or too high IBS or heterozygosity.  Removed people that did not pass sex chromosome checks or were < 18 years of age. | Corrected using mixed model regression analysis. | UA was measured with the colorimetric method using Targa 3000 from Biotecnica Instruments |
| Ogliastra Genetic Park(OGP) -Talana | Ethics Committee of the Italian Ministry Education, University and Research. | Population-based study with pedigree information | 860 | none | none | none | Uric acid levels were measured using the uricase method with an automated TARGA BT-3000 Chemistry Analyser |
| INCIPE | Institutional Ethics Committees | Randomly chosen from the lists of patients of 62 randomly selected general practitioners (GPs) based in four geographical areas in the Veneto region, Northern Italy. | 942 from Northern Italy | none | 992 genotyped individuals (then 50 removed). Disagreement between reported and genotypic sex, one randomly selected member of a pair of first-degree relatives | From same geographical area | UA was measured using the UV uricase method; the between series CV is 1.5% |
| New-Zealand sample  (NZPOLY) | New Zealand Ministry of Health Multiregional ethics Committee | Cross-sectional, population-based | 309 of self-reported Polynesian ancestry (NZ Maori and Pacific Island individuals) | none | none | none | UA was measured using the uricase oxidation method with a Roche chemistry modular P/D analyser at a commercial accredited laboratory. |
